# Whole-genome resequencing provides insights into the diversity and adaptation to desert environment in Xinjiang Mongolian cattle

**DOI:** 10.1186/s12864-024-10084-w

**Published:** 2024-02-14

**Authors:** Lei Xu, Kaiqing Zhou, Xixia Huang, Hong Chen, Hong Dong, Qiuming Chen

**Affiliations:** 1 College of Animal Science, Xinjiang Agricultural University, Urumqi, Xinjiang China; 2 Institute of Animal Science, Xinjiang Academy of Animal Science, Urumqi, Xinjiang China

## Abstract

**Background:**

Xinjiang Mongolian cattle is an indigenous breed that inhabits the Taklimakan Desert and is characterized by its small body size. However, the genomic diversity, origin, and genetic basis underlying the adaptation to the desert environment have been poorly studied.

**Results:**

We analyzed patterns of Xinjiang Mongolian cattle genetic variation by sequencing 20 genomes together with seven previously sequenced genomes and comparing them to the 134 genomes of nine representative breeds worldwide. Among the breeds of *Bos taurus*, we found the highest nucleotide diversity (0.0024) associated with the lower inbreeding coefficient (2.0110^-6^), the lowest linkage disequilibrium (r^2^ = 0.3889 at distance of 10 kb), and the highest effective population size (181 at 20 generations ago) in Xinjiang Mongolian cattle. The genomic diversity pattern could be explained by a limited introgression of *Bos indicus* genes. More importantly, similarly to desert-adapted camel and same-habitat sheep, we also identified signatures of selection including genes, GO terms, and/or KEGG pathways controlling water reabsorption and osmoregulation, metabolic regulation and energy balance, as well as small body size in Xinjiang Mongolian cattle.

**Conclusions:**

Our results imply that Xinjiang Mongolian cattle might have acquired distinct genomic diversity by virtue of the introgression of *Bos indicus*, which helps understand the demographic history. The identification of selection signatures can provide novel insights into the genomic basis underlying the adaptation of Xinjiang Mongolian cattle to the desert environment.

**Supplementary Information:**

The online version contains supplementary material available at 10.1186/s12864-024-10084-w.

Cattle, comprising two subspecies of *Bos taurus* and *Bos indicus*, can be traced back to early domestication events that occurred approximately 8,000–10,000 years ago in the Fertile Crescent and 6,000–8,000 years ago in the Indus Valley Cattle [1, 2]. Subsequent to that period, the interplay of natural adaptability, human migration, and selection has led to the emergence of more than 800 distinct breeds of cattle [3]. These breeds are distinguished by their diverse purposes, coat colors, geographical distributions, and other relevant aspects.

However, owing to variations in practice across different nations as well as disparities in significance, quantity, or other factors within the same country, the definition of certain breeds remains ambiguous. One illustrative instance of such a phenomenon is represented by Mongolian cattle, mostly found in the Inner Mongolia region of China, Mongolia, and the East Siberian regions of Russia. Based on a comprehensive investigation of whole-genome sequences, it was determined that the Mongolian cattle originating from Inner Mongolia of China and Mongolia were classified under the East Asian taurine group, clustering with the Mishima and Hanwoo cattle [4], also known as Turano-Mongolian taurine. At the level of the mitochondrial genome, Mongolian cattle exhibit the presence of four distinct haplotypes (T2, T3, T4, and I) [5], indicating intricate population dynamics.

Xinjiang Mongolian cattle, also referred to as Mongolian cattle, are primarily found in Bayangol Mongol of Xinjiang, China. The breed’s core regions are Hejin County and Heshuo County. It serves as a valuable source of milk and meat for the local Mongolian herdsmen. Xinjiang Mongolian cattle are characterized by their small body size, with females reaching a height at the withers of only 121 cm at the age of 15–16 months. Along with the implementation of genetic improvement via crossbreeding with other breeds like Simmental and Hereford cattle, the number of the breed has greatly decreased to about 6000. In terms of appearance, the coat color of Xinjiang Mongolian cattle is mixed, with a predominant yellow hue, followed by black. These cattle also possess crescent-shaped horns [6–8].

However, apart from the revelation of maternal (*Bos taurus* and *Bos indicus*) and paternal origin (*Bos taurus*) via SNP analysis of the mitochondrial DNA (mtDNA) D-loop region and Y chromosome [9], limited studies have focused on Xinjiang Mongolian cattle in molecular genetic research. On a global scale, analyses of whole-genome sequences of domesticated cattle have shown the presence of five to six ancestries, with some adaptive introgression events occurring during their migration from the domestication center to their current habitats [10–12].

Xinjiang Mongolian cattle are also known to inhabit the Taklimakan Desert, an environment characterized by arid and water-scarce conditions. Studies comparing the genomes of non-desert sheep breeds and desert sheep breeds in the same habitat as Xinjiang Mongolian cattle have identified a set of candidate genes responsible for water retention and reabsorption, ion transmembrane transport, and bicarbonate absorption [13]. Furthermore, in the context of adaptation to desert environments, the genetic mechanisms of camels have been extensively studied. Comparative genomic analyses have revealed the camel’s genetic adaptations to desert environments, including metabolic pathways such as water and salt metabolism, insulin resistance, oxidative stress responses, and osmoregulation [14–17], etc. Additionally, genomic studies of other desert-dwelling mammals have implicated genes related to regulation of thyroid levels [18], immune defense [19], response to radiation [20], body size [21].

In the present study, we sequenced the whole genomes of 20 Xinjiang Mongolian cattle and compared them to the public-available genomes of 141 samples of 10 breeds/populations (including seven genomes of Xinjiang Mongolian cattle). We not only identified the genomic diversity of Xinjiang Mongolian cattle, but also reconstructed the phylogenetic relationship between Xinjiang Mongolian cattle and other nine cattle breeds/populations to understand population dynamics of Xinjiang Mongolian cattle. More importantly, we delved into the selection signatures of Xinjiang Mongolian cattle by comparing the differences in nucleotide diversity, allele frequency and haplotype length to reveal the genetic basis of the adaptation to desert environment.

## Materials and methods

### Animal sampling

A total of 20 Xinjiang Mongolian cattle were obtained from the preserving farm after obtaining permission from farm owner, in Heshuo County of Bayangol Mongol Prefecture of Xinjiang in China. The whole blood was collected from the jugular vein of each individual with a blood collection needle by an experienced veterinarian and then placed in a blood collection tube containing EDTA.

### Genomic DNA extraction, genome sequencing, and SNP detection

The collected whole blood was delivered to the Beijing Genome Institute (Shenzhen, China) for DNA extraction, library construction and genome sequencing. The genomic DNA was extracted using the standard phenol-chloroform procedure. The concentration of genomic DNA was assessed by a Qubit fluorometer (Invitrogen, Carlsbad, USA), while the integrity of the DNA was verified using agarose gel electrophoresis. The qualified genomic DNA was randomly fragmented, and fragments with an average insert size of 313 bp were selected. Subsequently, the fragmented DNA underwent end-repair and 3′ adenylation. Adaptors were then ligated to the ends of these 3′-adenylated fragments. Following this, a PCR reaction and library quality control were performed to amplify the product and ensure it met the required standards.

The denatured single-stranded PCR products were subjected to a reaction system and program for circularization, resulting in the production of single-stranded cyclized products. Meanwhile, uncyclized linear DNA molecules were digested. The single-stranded circular DNA molecules were replicated using rolling cycle amplification, generating DNA nanoballs (DNBs) containing multiple copies of DNA. High-quality DNBs were then loaded into patterned nanoarrays using the high-intensity DNA nanochip technique and sequenced through combinatorial Probe-Anchor Synthesis (cPAS) using the DNBSEQ-T7 platform (BGI, Shenzhen, China), finally generating 2 × 150 bp pair-end reads. The sequenced raw reads were filtered using SOAPnuke software [22] with the parameters: “-n 0.01 -l 20 -q 0.3 --adaMR 0.25 --ada_trim --polyX 50”.

**Fig. 1:**
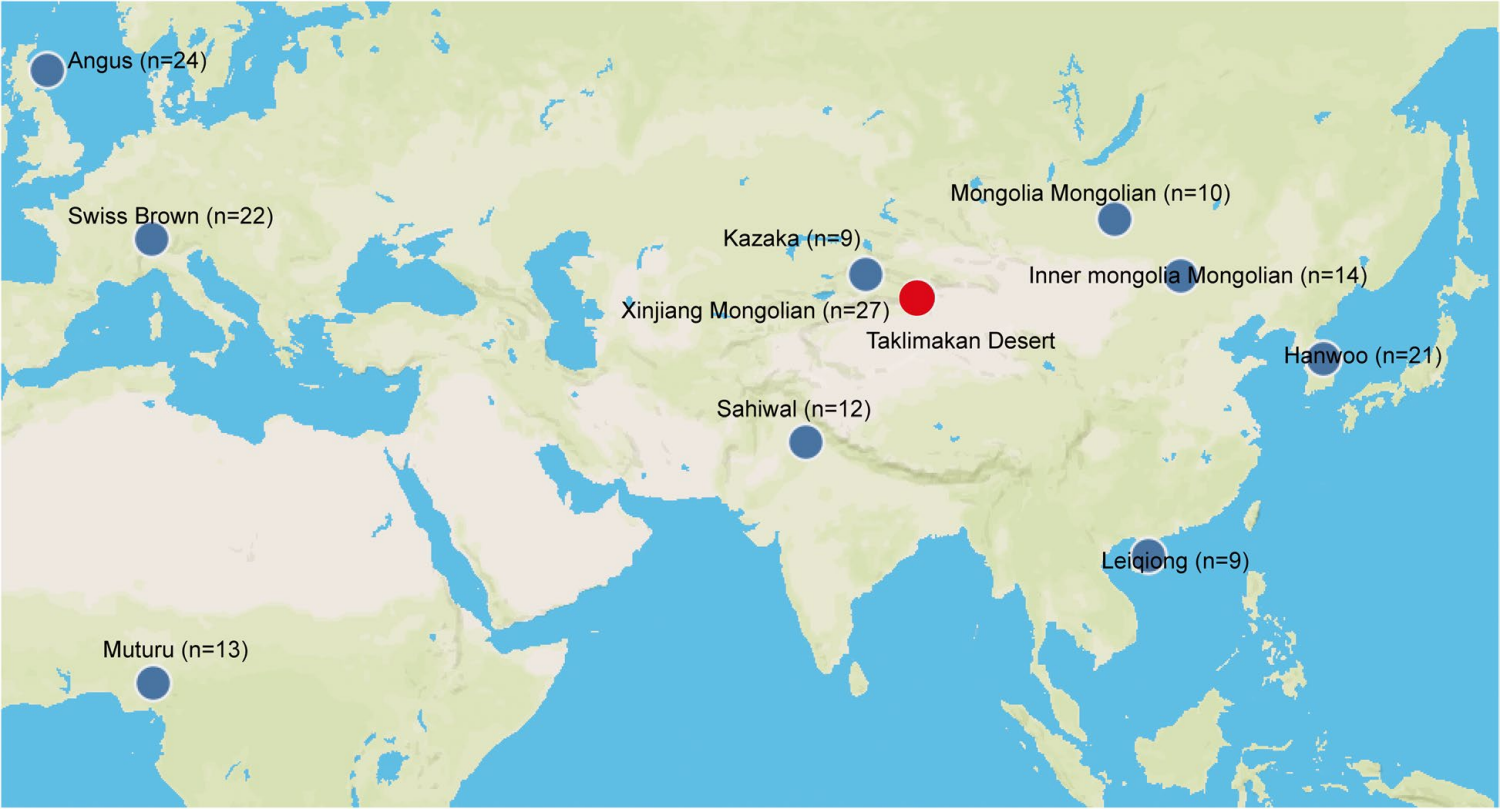
Geographic map illustrates the origins of the 10 cattle breeds/populations in the present study

To place Xinjiang Mongolian cattle into the global context of six ancestries worldwide reported by previous studies [11, 12] and to compare Xinjiang Mongolian cattle to other Mongolian cattle, we downloaded 141 public-available genomes of 10 breeds/populations, including 7 Xinjiang Mongolian cattle, 24 Angus (European taurine), 22 Swiss Brown (Eurasian taurine), 13 Muturu (African taurine), 9 Kazakh (another indigenous cattle in Xinjiang), 10 Mongolia Mongolian, 14 Inner Mongolia Mongolian, 21 Hanwoo (East Asian taurine), 12 Sahiwal (Indian indicine) and 9 Leiqiong cattle (Chinese indicine) from the NCBI SRA database (Fig. 1 and Supplementary Table 1). The downloaded reads were trimmed using Trimmomatic [23] with the following parameters: “LEADING:20 TRAILING:20 SLIDINGWINDOW:3:15 AVGQUAL:20 MINLEN:35 TOPHRED33”.

The clean reads were aligned to the *Bos taurus* assembly ARS-UCD1.2 using the BWA MEM algorithm [24] with the default parameters. The aligned reads were sorted on their coordinates and duplicates were marked using the SortSam and MarkDuplicates modules of the Picard software.

The BaseRecalibrator and PrintReads modules of GATK [25] were exploited to produce a base quality recalibration table and recalibrate the aligned BAM files. The HaplotypeCaller, CombineGVCFs, GenotypeGVCFs and SelectVariants modules of GATK were sequentially utilised for the purpose of SNP calling. The raw SNPs were filtered using VariantFiltration modules of GATK with the following parameters: “QD < 2.0||FS > 60.0||MQ < 40.0||MQRankSum < − 12.5||ReadPosRankSum < − 8.0||SOR > 3.0”.

#### Population structure and phylogenetic analysis

Principle component analysis (PCA) of whole-genome SNPs for two sets: all 161 individuals and 140 *Bos taurus* individuals, was performed using smartpca modules of EIGENSOFT [26]. A neighbor-joining (NJ) tree was constructed using MEGA11 [27] based on the Hamming distance obtained by PLINK with the --distance-matrix option. A maximum likelihood tree was constructed using treemix [28], where the number of anticipated migration events (m) varied from 0 to 10. The optimal number of assumed migration events was determined using the OptM method [29]. In addition, the process of estimating ancestry was conducted using the ADMIXTURE algorithm [30], where the range of presumed genetic clusters K varied from 2 to 10.

### General genomic characteristic

The nucleotide diversity of each breed was calculated using VCFtools [31], employing a window size of 50 Kb and a step size of 20 Kb. The identification of runs of homozygosity (ROH) in each individual was performed using PLINK [32] with the following parameters: “--homozyg --allow-no-sex --homozyg-window-snp 50 --homozyg-window-het 1 --homozyg-window-missing 5 --homozyg-density 50 --homozyg-kb 1000 --homozyg-snp 100”. The inbreeding coefficient based on runs of homozygosity (F_ROH_) was calculated for each individual as the ratio of the total length of ROH segments to the length of the autosomal genome. The average linkage disequilibrium (r^2^) for each length of distance for each breed was calculated using PopLDdecay [33]. In addition, the estimation of the recent effective population size (Ne) for each breed was conducted using SNeP [34] with the default settings.

### Detection of selection signatures

In order to elucidate the genomic patterns of adaptation to desert environments, we employed three different methodologies to identify selection signatures in Xinjiang Mongolian cattle. This involved comparing the genomes of Xinjiang Mongolian cattle to those of Mongolia Mongolian and Inner Mongolia Mongolian cattle, which served as the control group.

The first approach was the calculation of the nucleotide diversity (π) ratio derived by dividing the nucleotide diversity of the control group by that of Xinjiang Mongolian cattle, in which the π of Xinjiang Mongolian cattle and its counterpart was computed using VCFtools with the windows of 50 kb and steps of 20 kb.

The second approach was the measurement of the genetic differentiation between Xinjiang Mongolian cattle and the control group (*F*_*ST*_), whose genome-wide distribution was determined using VCFtools with the same window and step size as the π.

Third, the clean SNPs underwent further filtering using VCFtools with the following parameters: “--min-alleles 2 --max-alleles 2 --max-missing 0.9”. Subsequently, the imputation and phasing processes were carried out using Beagle [35]. The cross-population extended haplotype homozygosity (XP-EHH) score for every site was calculated using selscan and then was normalized using the norm module of selscan [36]. The average normalized XP-EHH score for each 50 kb region with 20 kb increments was defined as the XP-EHH statistic, which was the third algorithms.

Finally, the outlier windows supported by two or three approaches (top 1%) were defined as candidate regions under positive selection and then annotated using a custom perl script based on the annotation GFF file of the *Bos taurus* genome (ARS-UCD1.2). Based on the candidate genes under selection, the functional enrichment tests of the Kyoto Encyclopaedia of Genes and Genomes (KEGG) pathways and Gene Ontology (GO) database were performed using the DAVID server [37]. For multiple testing of the enrichment analysis, the Benjamini-Hochberg false discovery rate (FDR) was applied.

## Results

### Summary of sequencing and SNP detection

Whole-genome sequencing of 20 samples of Xinjiang Mongolian cattle generated a total of 4.7 billion reads with an average depth of 12.80 (12.76–12.84). The average sequence depth was 11.56 (10.45–12.33) for seven publicly available genomes of Xinjiang Mongolian cattle and 12.48 (4.91–39.04) for the remaining 134 publicly available genomes of nine breeds/populations (Supplementary Table 1).

Combining the genomes of Xiangjiang Mongolian cattle and other nine breeds/populations, a total of 61,528,133 SNPs were identified. The majority of the SNPs were found in the intergenic region (59.37%), followed by intronic regions (37.22%), while only a small number of the SNPs (1.14%), with 255,920 non-synonymous SNPs and 425,549 synonymous SNPs, were found in exonic regions (Supplementary Table 2).

## Population structure

**Fig. 2:**
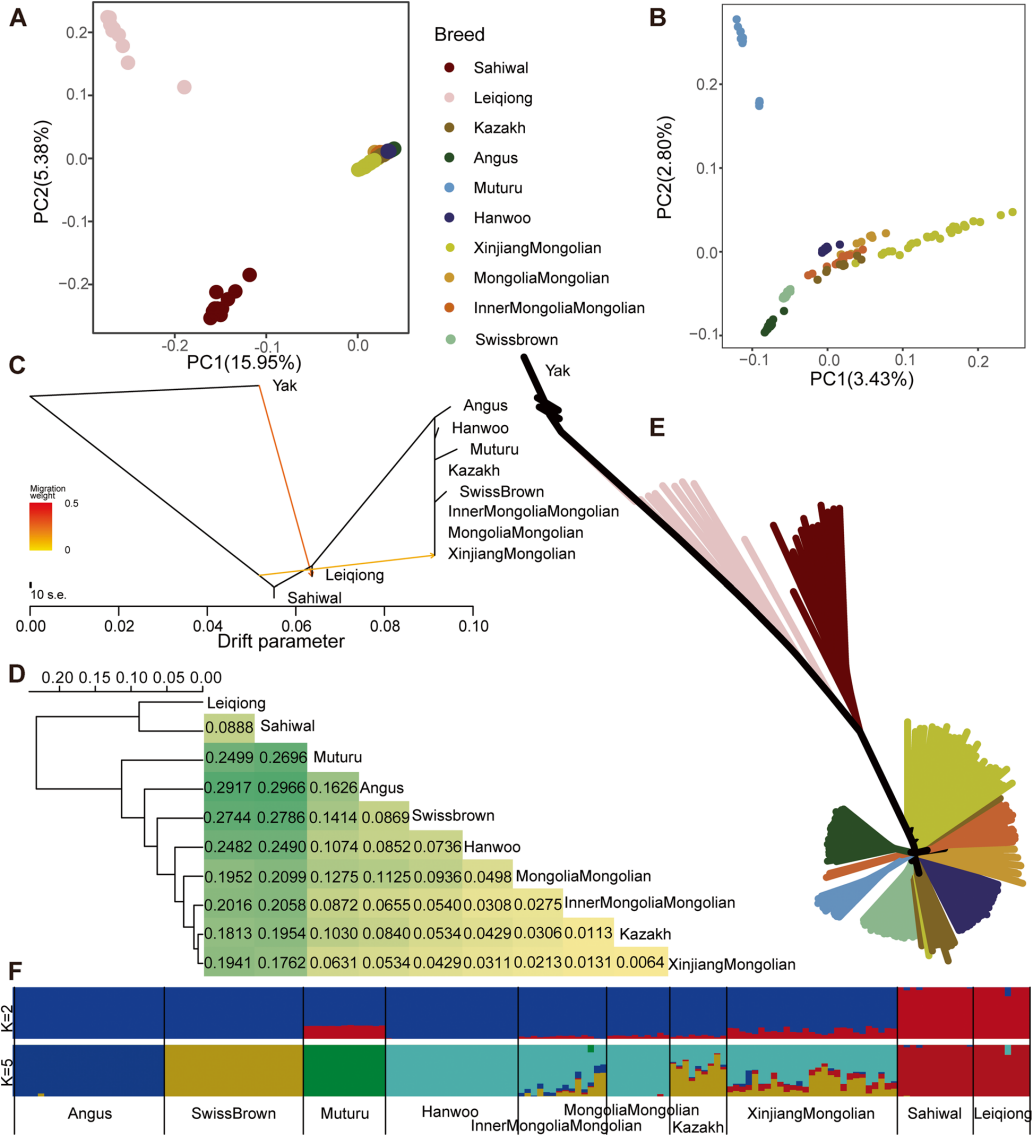
Phylogenetic relationship of the 10 cattle breeds. **A** Plots of the first and the second principal components for all the 161 individuals. **B** Plots of the first and the second principal components for the 140 taurine individuals. **C** Maximum likelihood tree was constructed with TreeMix using the best two migration events supported by *OptM*. **D** Breed hierarchical clustering (left) and heatmap (right) based on the genetic differentiation (*F*_*ST*_). **E** Neighbor-joining tree of the relationships of the 161 individuals was constructed based on Hamming genetic distances matrix. **F** Proportion of ancestry components for all the 161 individuals was determined using the ADMIXTURE model with K = 2 and K = 5. K = 5 is a sensible modeling choice of the standard error of the cross-validation error

PCA for all 161 samples revealed the first principal component (PC1) and the second principal component (PC2), explaining 15.95% and 5.38% of the total variation, respectively. This analysis successfully distinguished *Bos taurus*, Leiqiong, and Sahiwal cattle (Fig. 2A). Within the *Bos taurus* subspecies, the first two principal components (PCs) explained 3.43% and 2.80% of the overall variation, respectively. These PCs primarily reflected distinctions among three distinct clusters: European commercial breeds (Angus and Swiss Brown cattle), Xinjiang Mongolian cattle, and African Muturu cattle. The remaining four breeds/populations occupied an intermediate position in this regard (Fig. 2B). The maximum likelihood tree with the best number of migration events (Supplementary Fig. 1) provided evidence for the gene flow from *Bos indicus* into Xinjiang Mongolian cattle (Fig. 2C). Meanwhile, the genetic differentiation (*F*_*ST*_) observed between Xinjiang Mongolian cattle and *Bos indicu*s was comparatively smaller when compared to the differentiation shown between other *Bos taurus* populations and *Bos indicus* (Fig. 2D). The NJ tree displayed two distinct groups, namely *Bos taurus* and *Bos indicus*. Among the *Bos taurus* group, Xinjiang Mongolian cattle had the closest genetic connection with *Bos indicus* (Fig. 2E). The ADMIXTURE analysis recapitulated the divergence of *Bos taurus* and *Bos indicus*, and a small proportion of indicine ancestry in our Xinjiang Mongolian cattle (around 16%.

on average) at K = 2. At K = 5, the most reasonable biological interpretation (Supplementary Fig. 2), Xinjiang Mongolian, Inner Mongolia Mongolian and Kazakh cattle have four genomic compositions, and among these three populations, Xinjiang Mongolian cattle demonstrate the highest degree of *Bos indicus* inheritance (Fig. 2F).

### Genome diversity

**Fig. 3:**
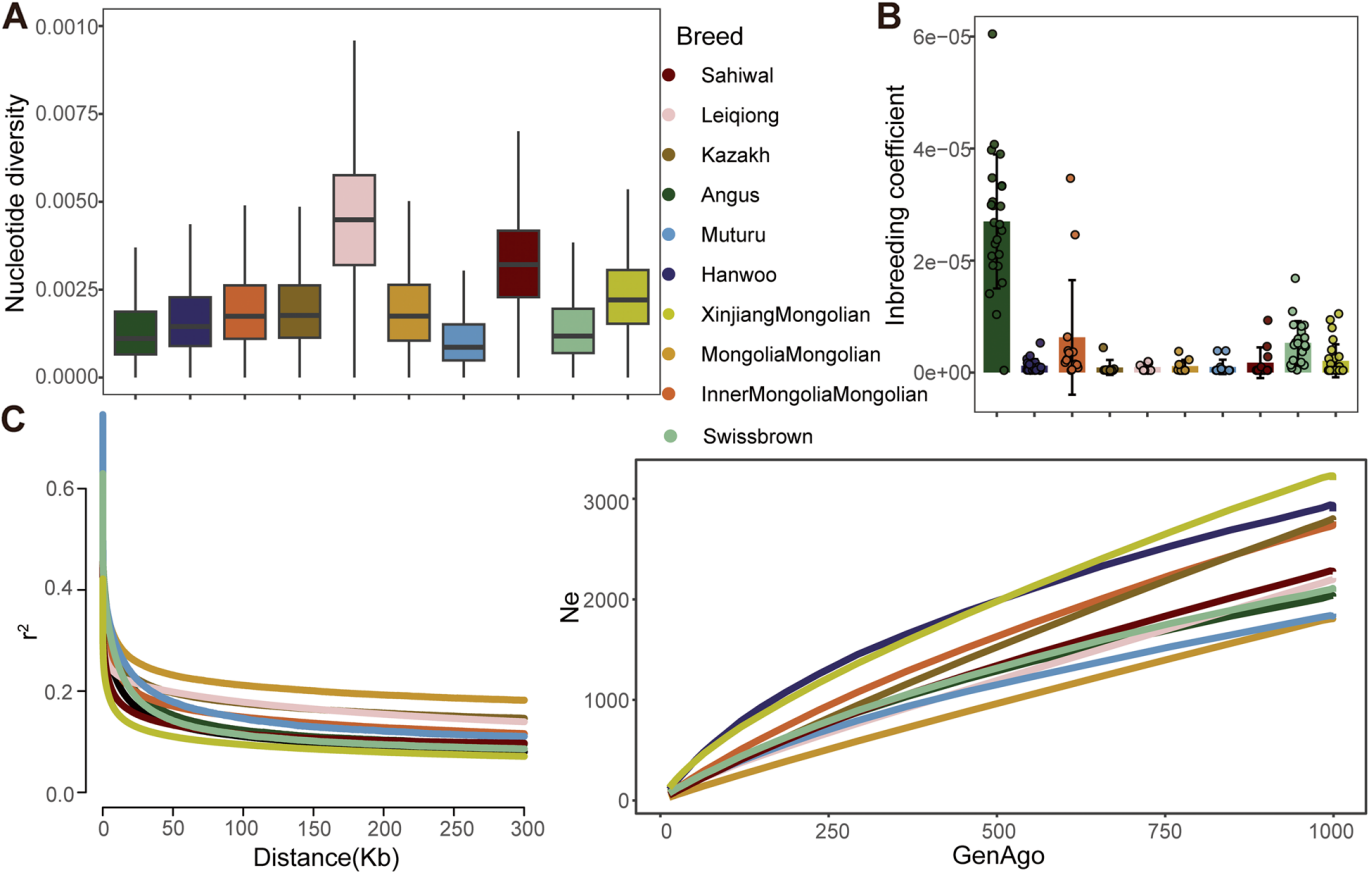
Genomic characteristics of the 10 cattle breeds. **A** Box plots of nucleotide diversity for each breed in 50 kb sliding windows with 20 kb steps. The boxplots were made using ggplot2, and the outliers were not shown. **B** ROH-based inbreeding coefficient (*F*_ROH_) on cattle autosomes estimated from each individual. **C** Decay of Linkage disequilibrium (r^2^) was assessed for each breed. **D** The recent effective population size was estimated for each breed

The highest nucleotide diversity was observed in *Bos indicus* (Leiqiong and Sahiwal cattle), followed by three Mongolian cattle populations, Kazakh and Hanoo cattle, while the lowest nucleotide diversity was observed in two commercial *Bos taurus* (Swiss Brown and Angus cattle) and Muturu cattle (Fig. 3A). In ROH, the *Bos indicus* breeds showed the lowest inbreeding coefficient, while two commercial *Bos taurus* showed the highest inbreeding coefficient, other six breeds/populations are in the middle (Fig. 3B). LD analysis showed that the highest average LD (r^2^) was found in Muturu cattle, followed by two commercial *Bos taurus* breeds, Mongolia Mongolian, Inner Mongolia Mongolian, Kazakh, Sahiwal and Hanwoo cattle, while the lowest average LD was found in Leiqiong and Xinjiang Mongolian cattle at the time of the short distance between SNPs. The LD analysis also showed the fastest decay in the two commercial *Bos taurus* breeds (Fig. 3C). In addition, the highest Ne was found in Xinjiang Mongolian and Hanwoo cattle, while the lowest Ne was found in Sahiwal and Mongolia Mongolian cattle in the recent generations ago (Fig. 3D).

### Selection signatures in Xinjiang Mongolian cattle

**Fig. 4:**
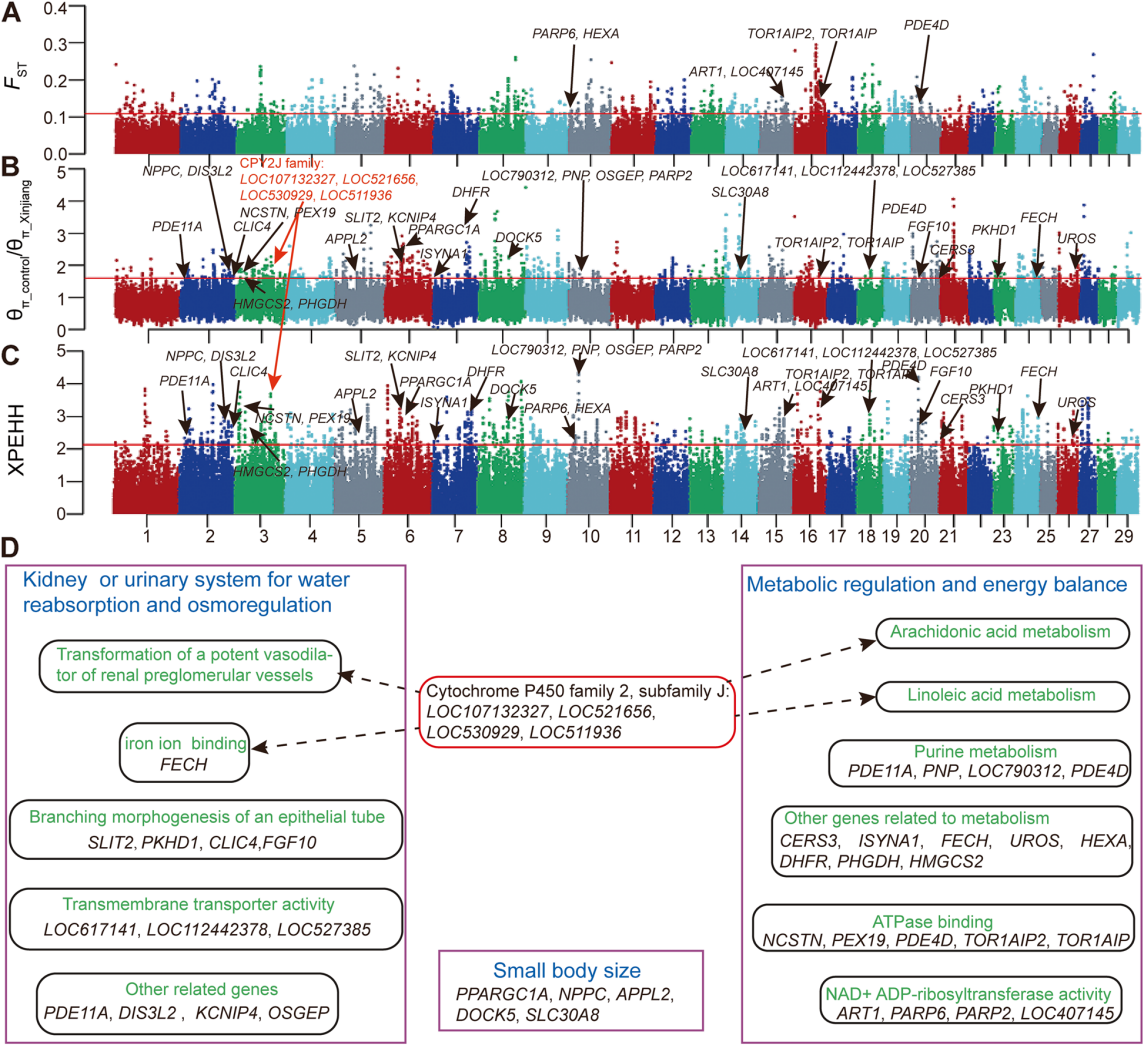
Genomic regions with selection signatures in Xinjiang Mongolian cattle. Genome-wide distribution of the π ratio (**A**), *F*_*ST*_ (**B**) and XP-EHH (**C**). The red line represents the threshold value for the top 1% based on three metrics: the π ratio (1.61), *F*_*ST*_ (0.11), and XP-EHH (2.13). **D** Functional annotation of several candidate selected genes responsible for the adaptation to desert environment. The names of the KEGG pathways, GO terms, or other functions are shown in green. The candidate genes positively selected are shown in black. The potential mechanisms behind the adaptation to desert environment are shown in blue. Four genes belonging to the cytochrome P450 family 2, subfamily J are implicated in various functions

To identify the genomic regions under selection in Xinjiang Mongolian cattle, we compared their genomes to the genomes of Mongolia Mongolian and Inner Mongolian cattle using the π ratio, *F*_*ST*_, and XP-EHH. After merging consecutive outlier windows supported by two or three approaches, a total of 94 unique regions containing 130 genes were identified (Fig. 4A, B and C, Supplementary Table 3).

In the functional enrichment analysis of the 130 candidate selected genes, we identified four over-represented GO terms (FDR < 0.05). The most significant GO term was oxidoreductase activity. Other significant enrichments included ATPase binding, NAD + ADP-ribosyltransferase activity, and steroid hydroxylase activity. Genes related to branching morphogenesis of an epithelial tube, protein ADP-ribosylation, L-lysine transmembrane transporter activity, arginine transmembrane transporter activity, L-ornithine transmembrane transporter activity, and iron ion binding were also enriched (Supplementary Table 4). In addition, the enrichment analysis of the KEGG pathway was performed, resulting in four metabolic related pathways in the top 10 significant categories (Supplementary Table 5), including linoleic acid metabolism, arachidonic acid metabolism, metabolic pathways, and purine metabolism.

Among 94 putatively selected regions, the cytochrome P450 family involved in oxidoreductase activity showed an extreme π ratio and XP-EHH, including four genes (*LOC107132327*, *LOC521656*, *LOC530929*, *LOC511936*). Other noteworthy genes with selection signatures included those with water reabsorption and osmoregulation (*PDE11A*, *DIS3L2*, *SLIT2*, *KCNIP4*, *OSGEP*, *PKHD1*, *LOC617141*, *LOC112442378*, *LOC527385*, *FECH*, *LOC107132327*, *LOC521656*, *LOC530929*, *LOC511936*, *CLIC4*, *FGF10*), metabolic regulation and energy balance (*NCSTN*, *PEX19*, *PDE4D*, *TOR1AIP2*, *TOR1AIP1*, *ART1*, *PARP6, PARP2*, *LOC407145*), and small body size (*PPARGC1A*, *NPPC*, *APPL2*, *DOCK5*, *SLC30A8*) (Fig. 4D).

## Discussion

In this study, we compared the genomes of 27 Xinjiang Mongolian cattle (20 resequenced and seven publicly available genomes) to the 134 genomes of nine representative breeds worldwide. Our exploration of the cattle genetic variations and selection signatures focused specifically on genomic diversity, breed origin, and candidate selected genes responsible for the adaptation to the desert environment in Xinjiang Mongolian cattle.

The findings from the PCA and NJ tree indicate that our Xinjiang Mongolian cattle clustered with Angus cattle, a breed with single *Bos taurus* ancestry [10, 11, 38, 39], and clearly separated from Sahiwal cattle, a breed with single *Bos indicus* ancestry [10, 11, 39]. In-depth analyses on ancestry proportion estimation and migration events showed clear evidence of genetic heterogeneity with small genomic composition with *Bos indicus* (Sahiwal cattle) in Xinjiang Mongolian cattle. This observation is supported by the lowest level of genetic differentiation observed in the Xinjiang Mongolian cattle when compared to the relationships between *Bos taurus* populations and *Bos indicus*. Moreover, the genetic differentiation between Xinjiang Mongolian and Sahiwal cattle was lower than that between Xinjiang Mongolian and Leiqiong cattle, a *Bos indicus* breed with introgression from other bovine species [10, 11], suggesting that the *Bos indicus* ancestry observed in Xinjiang Mongolian cattle was probably derived from Indian indicine rather than Chinese Southern indicine, which was consistent with previous inference from mitochondrial SNPs [9].

Compared with other *Bos taurus* breeds, our Xinjiang Mongolian cattle have the highest nucleotide diversity associated with the lower inbreeding coefficient, the lowest linkage disequilibrium, and the highest effective population size with the exception of a period between 54 and 454 generations ago. This unique diversity pattern could be explained by the *Bos taurus* group of Xinjiang Mongolian cattle with small introgression from *Bos indicus*. The phenomenon of increased diversity introduced from other ancestry was also reported in other breeds [10] even species [40]. In addition, we also observed the highest nucleotide diversity of *Bos indicus* (Sahiwal and Leiqiong cattle) with lower artificial selection or introgression from different bovine species [10], the faster LD decay of commercial European taurine with stronger artificial selection suggested by the highest inbreeding coefficient [4] and the lowest nucleotide diversity of African endangered Muturu cattle [41].

The most crucial attribute of Xinjiang Mongolian cattle is their adaptability to the desert environment. The identification of candidate genes under selection is of significant importance in elucidating the genetic basis underlying the well-adapted characteristics of Xinjiang Mongolian cattle residing in desert environments. Among the candidate selected genes identified by two or three approaches, functional enrichment analysis showed the most significance of oxidoreductase activity, consisting of four genes of the cytochrome P450 family 2, subfamily J. The cytochrome P450 enzymes, a type of monooxygenase with a prosthetic group of heme-iron, are involved in the metabolism of arachidonic acid, the secretion of the final metabolites in urine or bile, and the transformation of a potent vasodilator of renal preglomerular vessels stimulating water reabsorption [42]. Among these families, the CYP2J2 activity has been demonstrated to be regulated by high-salt diet and its suppression could result in hypertension [43]. It is noteworthy that in camel whose most distinctive feature is the adaptation to the desert environment, the number of the copies of CYP2J is higher than that in cattle, horse and human [14–16]. In fact, other members of cytochrome P450 family were also selected in other species in desert regions [44, 45]. The above studies indicate that variations in CYP2J subfamily genes may play an important in the adaptation of Xinjiang Mongolian cattle to the desert environment.

As has been said, kidneys play a major role in the process of water reabsorption through increasing the osmolarity of urine. We surveyed published literature and identified several selected genes associated with the kidney or urinary system. *PDE11A* gene along with extreme π ratio and XP-EHH encodes a dual-specificity phosphodiesterase and is expressed in adrenal cortex. It has been reported that a germline mutation at *PDE11A* locus is associated with adrenocortical hyperplasia in human [46]. *DIS3L2* gene also along with extreme π ratio and XP-EHH, encodes an exoribonuclease, and its germline mutations caused the Perlman syndrome with kidney abnormalities [47]. Among the selection candidates in our list are *SLIT2* (a ligand in ureteric bud and metanephric mesenchyme) in which the mutations confer for congenital anomalies of the kidney and urinary tract in human [48], *KCNIP4* a potassium channel-interacting protein whose translocation implicates renal cell cancer [49], *OSGEP* enabling N(6)-L-threonylcarbamoyladenine synthase activity and metal ion binding activity and associated with a renal defect manifesting in proteinuria and hypomagnesemia [50], *CLIC4* a cytosolic protein in which null mouse embryos exhibit impaired renal tubulogenesis [51], *FGF10* a multifunctional FGF family member implicated renal ischemia/reperfusion injury [52] and ureter and kidney development [53], and *PKHD1* a large transmembrane protein fibrocystin involved in polycystic kidney disease [54]. Notably, the GO term of branching morphogenesis of an epithelial tube (*PKHD1*, *CLIC4*, *SLIT2, FGF10*) was enriched, which is critical to ureteric bud formation and epithelial branching during kidney development [55]. Moreover, the enrichment analysis also showed three GO terms (including three genes *LOC617141*, *LOC112442378*, *LOC527385*) associated with transmembrane transporter activity involved in osmoregulation in the renal medulla in camel [17]. In addition, an overrepresentation of categories associated with iron ion binding was also detected, including five genes *FECH*, *LOC107132327*, *LOC521656*, *LOC530929*, *LOC511936*). In fact, similar GO terms were also detected in enrichment analysis of differentially expressed genes between water-restricted and normal condition in renal cortical and medullary of bactrian camel [17]. Similarly, an excellent study also revealed several selected genes related to renal vasodilation, ion transmembrane transport, water-salt metabolism, and bicarbonate absorption in Taklimakan Desert sheep [13]. From the above results, we can conclude that these genes could have been affected by selection targeting at water reabsorption and osmoregulation during the adaptation to desert environment.

The scarcity of food in desert environments presents a secondary barrier to adaptation, hence emphasizing the significance of energy and metabolism. The second-ranked GO term identified by enrichment analysis is ATPase binding, which is related to catalyze the hydrolysis of ATP, a key player in biological energy capture and use, including five genes (*NCSTN*, *PEX19*, *PDE4D*, *TOR1AIP2*, *TOR1AIP1*). The third-ranked GO term is NAD + ADP-ribosyltransferase activity involved in cellular energy levels [56], including four genes (*ART1*, *PARP6, PARP2*, *LOC407145*). Moreover, four of the top 10 KEGG pathways identified by enrichment analysis are related to metabolism, and one of them is arachidonic acid metabolism which is also implicated in adaptation to desert environments in camel [16] and sheep [13], including four genes (*LOC107132327*, *LOC521656*, *LOC530929*, *LOC511936*). Therefore, we could speculate that these genes, GO terms, or pathways may play a role in metabolic regulation and energy balance in desert environment adaptations.

For animals living in extreme environments, small body size could help overcome the challenge of scarce food supply by virtue of lower metabolic requirement and less energy consumption [13, 57]. After reviewing published literature on candidate selected genes, a total of five genes were found to be associated with body size traits. One interesting observation was the presence of *PPARGC1A* with strong signature of selection. *PPARGC1A* encodes a transcriptional coactivator that regulates the genes involved in energy metabolism and plays an important role in insulin signaling, mitochondrial regulation and adaptive thermogenesis [58–60]. Its mutations have been reported to be associated with body mass index in human [61]. Another candidate gene is *NPPC*, a natriuretic peptide that regulates endochondral ossification of the cartilaginous selected growth plate, in which SNP markers are associated with human height [62]. Other noteworthy genes in our candidate list were *APPL2*, an adiponectin receptor associated with overweight and obesity in a Chinese population [63], *DOCK5*, a susceptibility gene for severe obesity [64], and *SLC30A8*, a zinc efflux transporter implicated in type 2 diabetes and obesity in Asians [65]. These genes together may be under selection for the reduced body size of Xinjiang Mongolian in the adaptation to the desert environment.

## Conclusions

This study reports the current genetic status and novel insights on the adaptation of Xinjiang Mongolian cattle in the Taklimakan Desert using whole-genome sequence data. The unique diversity pattern observed advances our understanding of historical population dynamics in Xinjiang Mongolian cattle. In a similar manner to camels and sheep that reside in desert environments, selection signatures for genes involved in the regulation of water reabsorption, energy balance, and small body size are indicative of their heightened adaptation to desert environments. This observation offers valuable insights into the genetic mechanisms that underlie the process of environmental adaptation.

### Supplementary Information

**Additional file 1.** **Supplementary Figure 1.** The output produced by *OptM*. A total of 5 iterations were run for each possible number of migration edges, *m* = 1–10. (**A**) The mean and standard deviation (SD) for the composite likelihood *L*(*m*) (left axis, black circles) and proportion of variance explained (right axis, red “x”s). (**B**) The second-order rate of change (Δ*m*) across values of *m*. The arrow indicates the peak in Δ*m* at *m* = 2 edges. **Supplementary Figure 2. **Cross-validation plot for the 161 genomes. **Supplementary Table 1. **Summary of sequencing data. **Supplementary Table 2. **Functional classification of the detected SNPs. **Supplementary Table 3. **Functional classification of the exonic SNPs. **Supplementary Table 4. **list of selected regions in Xinjiang Mongolian cattle. **Supplementary Table 5. **The top ten significant GO terms from the enrichment analysis of selected candidate genes **Supplementary Table 6. **The top ten significant KEGG pathways from the enrichment analysis of selected candidate genes.

## Data Availability

Whole genome sequencing data of Xinjiang Mongolian cattle have been successfully submitted to the National Center for Biotechnology Information. SRA data: PRJNA844484.
