# Supplementary material for: Whole-genome resequencing provides insights into the diversity and adaptation to desert environment in Xinjiang Mongolian cattle
Source: BMC Genomics. 2024 Feb 14;25:176. doi: 10.1186/s12864-024-10084-w (PMC10865613; doi:10.1186/s12864-024-10084-w)
Supplement: Supplementary file 1 — Additional file 1. Supplementary Figure 1. The output produced by OptM. A total of 5 iterations were run for each possible number of migration edges, m = 1–10. (A) The mean and standard deviation (SD) for the composite likelihood L(m) (left axis, black circles) and proportion of variance explained (right axis, red “x”s). (B) The second-order rate of change (Δm) across values of m. The arrow indicates the peak in Δm at m = 2 edges. Supplementary Figure 2. Cross-validation plot for the 161 genomes. Supplementary Table 1. Summary of sequencing data. Supplementary Table 2. Functional classification of the detected SNPs. Supplementary Table 3. Functional classification of the exonic SNPs. Supplementary Table 4. list of selected regions in Xinjiang Mongolian cattle. Supplementary Table 5. The top ten significant GO terms from the enrichment analysis of selected candidate genes Supplementary Table 6. The top ten significant KEGG pathways from the enrichment analysis of selected candidate genes. [file 12864_2024_10084_MOESM1_ESM.docx]

**Whole-genome resequencing provides insights into the diversity and adaptation to desert environment in Xinjiang Mongolian cattle**


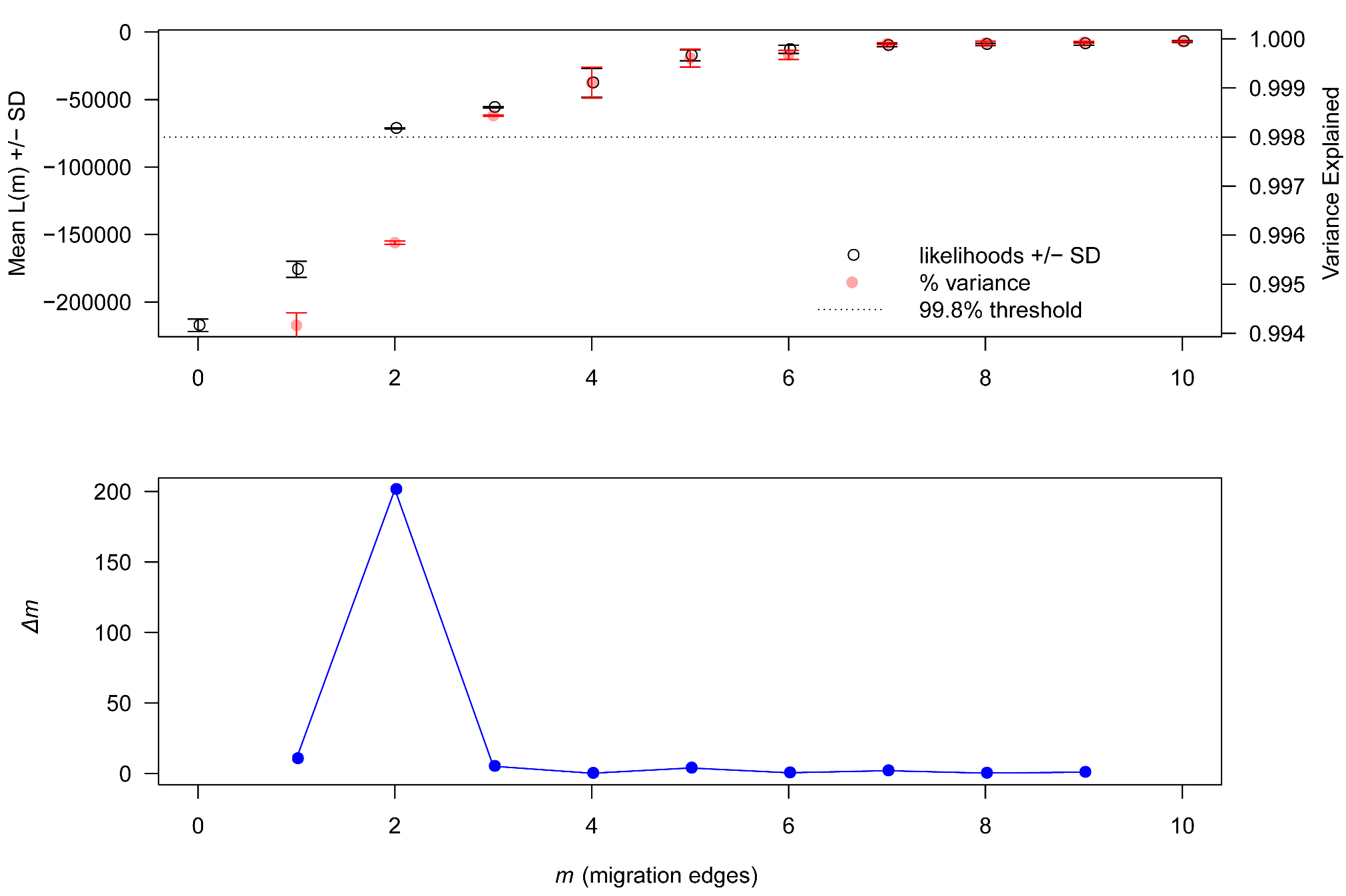


**Supplementary Figure 1** The output produced by OptM. A total of 5 iterations were run for each possible number of migration edges, m = 1–10. (**A**) The mean and standard deviation (SD) for the composite likelihood L(m) (left axis, black circles) and proportion of variance explained (right axis, red “x”s). (**B**) The second-order rate of change (Δm) across values of m. The arrow indicates the peak in Δm at m = 2 edges.


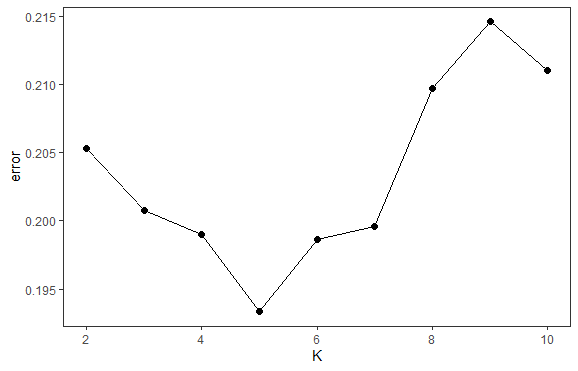


**Supplementary Figure 2** Cross-validation plot for the 161 genomes.

**Supplementary Table 1** Summary of sequencing data.

| accession Number | BioProject | sample Number | Breed | Number of clean reads | Number of Aligned reads | Mapping rate | Number of bases | Mean depth | Selected reason |
| --- | --- | --- | --- | --- | --- | --- | --- | --- | --- |
| ERR1746315 | PRJEB18113 | SAMEA19314418 | SwissBrown | 339,562,799 | 338,267,701 | 99.62% | 44,407,739,624 | 16.0999X | European taurine |
| ERR2985355 | PRJEB18113 | SAMEA5159769 | SwissBrown | 340,079,112 | 336,416,283 | 98.92% | 33,394,855,655 | 12.1069X | European taurine |
| ERR2985356 | PRJEB18113 | SAMEA5159770 | SwissBrown | 276,364,827 | 272,678,114 | 98.67% | 26,981,228,930 | 9.7816X | European taurine |
| ERR2985357 | PRJEB18113 | SAMEA5159771 | SwissBrown | 300,537,982 | 297,385,186 | 98.95% | 29,568,244,461 | 10.7196X | European taurine |
| ERR2985358 | PRJEB18113 | SAMEA5159772 | SwissBrown | 329,607,676 | 326,114,889 | 98.94% | 32,330,316,744 | 11.7209X | European taurine |
| ERR2985359 | PRJEB18113 | SAMEA5159773 | SwissBrown | 297,648,567 | 294,049,136 | 98.79% | 29,214,959,145 | 10.5916X | European taurine |
| ERR2985361 | PRJEB18113 | SAMEA5159775 | SwissBrown | 324,185,264 | 321,292,233 | 99.11% | 30,542,374,023 | 11.0725X | European taurine |
| ERR2985365 | PRJEB18113 | SAMEA5159779 | SwissBrown | 300,279,933 | 299,480,853 | 99.73% | 29,374,577,090 | 10.6492X | European taurine |
| ERR2985366 | PRJEB18113 | SAMEA5159780 | SwissBrown | 348,750,996 | 344,136,664 | 98.68% | 33,910,649,825 | 12.2937X | European taurine |
| ERR2985367 | PRJEB18113 | SAMEA5159781 | SwissBrown | 358,741,603 | 352,635,227 | 98.30% | 34,713,379,195 | 12.5848X | European taurine |
| ERR2985368 | PRJEB18113 | SAMEA5159782 | SwissBrown | 320,204,241 | 316,238,068 | 98.76% | 31,271,473,353 | 11.337X | European taurine |
| ERR2985369 | PRJEB18113 | SAMEA5159783 | SwissBrown | 333,752,118 | 329,604,851 | 98.76% | 32,524,265,904 | 11.7911X | European taurine |
| ERR2985370 | PRJEB18113 | SAMEA5159784 | SwissBrown | 332,744,470 | 329,000,958 | 98.87% | 32,466,259,625 | 11.7701X | European taurine |
| ERR2985371 | PRJEB18113 | SAMEA5159785 | SwissBrown | 336,672,744 | 331,549,481 | 98.48% | 32,699,508,171 | 11.8546X | European taurine |
| ERR2985372 | PRJEB18113 | SAMEA5159786 | SwissBrown | 339,572,694 | 334,546,680 | 98.52% | 33,009,137,243 | 11.9669X | European taurine |
| ERR2985374 | PRJEB18113 | SAMEA5159788 | SwissBrown | 345,273,293 | 305,314,364 | 88.43% | 29,718,855,342 | 10.774X | European taurine |
| ERR2985377 | PRJEB18113 | SAMEA5159791 | SwissBrown | 291,410,362 | 288,664,738 | 99.06% | 28,290,166,169 | 10.256X | European taurine |
| ERR2985378 | PRJEB18113 | SAMEA5159792 | SwissBrown | 304,789,151 | 301,808,093 | 99.02% | 29,580,176,260 | 10.7237X | European taurine |
| ERR2985379 | PRJEB18113 | SAMEA5159793 | SwissBrown | 362,611,805 | 341,870,988 | 94.28% | 33,919,061,012 | 12.2969X | European taurine |
| ERR2985383 | PRJEB18113 | SAMEA5159797 | SwissBrown | 346,379,953 | 343,864,894 | 99.27% | 33,481,038,931 | 12.1379X | European taurine |
| ERR2985384 | PRJEB18113 | SAMEA5159798 | SwissBrown | 377,235,282 | 374,046,108 | 99.15% | 36,143,404,253 | 13.103X | European taurine |
| ERR2985385 | PRJEB18113 | SAMEA5159799 | SwissBrown | 304,951,200 | 290,370,495 | 95.22% | 28,352,862,105 | 10.28X | European taurine |
| MG1 | OurStudy | MG1 | XinjiangMongolian | 237,365,111 | 236,881,496 | 99.80% | 35,398,145,705 | 12.8351 | Our focus |
| MG10 | OurStudy | MG10 | XinjiangMongolian | 236,950,742 | 235,571,454 | 99.42% | 35,199,946,959 | 12.7632 | Our focus |
| MG11 | OurStudy | MG11 | XinjiangMongolian | 237,172,552 | 236,688,654 | 99.80% | 35,372,227,219 | 12.8255 | Our focus |
| MG14 | OurStudy | MG14 | XinjiangMongolian | 236,796,690 | 235,623,060 | 99.50% | 35,184,030,830 | 12.7573 | Our focus |
| MG16 | OurStudy | MG16 | XinjiangMongolian | 237,213,694 | 236,458,340 | 99.68% | 35,332,931,609 | 12.8112 | Our focus |
| MG19 | OurStudy | MG19 | XinjiangMongolian | 237,518,382 | 237,054,510 | 99.80% | 35,411,920,187 | 12.8402 | Our focus |
| MG21 | OurStudy | MG21 | XinjiangMongolian | 236,954,519 | 236,117,348 | 99.65% | 35,255,351,336 | 12.7834 | Our focus |
| MG24 | OurStudy | MG24 | XinjiangMongolian | 236,788,312 | 236,466,566 | 99.86% | 35,327,123,823 | 12.8093 | Our focus |
| MG26 | OurStudy | MG26 | XinjiangMongolian | 236,963,346 | 236,197,592 | 99.68% | 35,286,200,368 | 12.7945 | Our focus |
| MG27 | OurStudy | MG27 | XinjiangMongolian | 236,920,500 | 236,432,107 | 99.79% | 35,331,352,939 | 12.811 | Our focus |
| MG28 | OurStudy | MG28 | XinjiangMongolian | 236,868,601 | 235,907,338 | 99.59% | 35,253,256,295 | 12.7825 | Our focus |
| MG3 | OurStudy | MG3 | XinjiangMongolian | 236,567,279 | 235,711,348 | 99.64% | 35,212,350,626 | 12.7677 | Our focus |
| MG31 | OurStudy | MG31 | XinjiangMongolian | 237,013,215 | 236,665,890 | 99.85% | 35,363,346,524 | 12.8226 | Our focus |
| MG33 | OurStudy | MG33 | XinjiangMongolian | 237,045,964 | 236,727,721 | 99.87% | 35,215,483,066 | 12.7688 | Our focus |
| MG34 | OurStudy | MG34 | XinjiangMongolian | 235,925,723 | 235,571,390 | 99.85% | 35,202,284,602 | 12.7638 | Our focus |
| MG35 | OurStudy | MG35 | XinjiangMongolian | 237,278,850 | 236,992,173 | 99.88% | 35,406,575,481 | 12.8378 | Our focus |
| MG36 | OurStudy | MG36 | XinjiangMongolian | 237,236,751 | 236,880,927 | 99.85% | 35,385,813,587 | 12.8304 | Our focus |
| MG37 | OurStudy | MG37 | XinjiangMongolian | 236,620,817 | 236,273,652 | 99.85% | 35,275,459,277 | 12.7907 | Our focus |
| MG4 | OurStudy | MG4 | XinjiangMongolian | 237,519,957 | 236,785,650 | 99.69% | 35,382,193,693 | 12.8294 | Our focus |
| MG9 | OurStudy | MG9 | XinjiangMongolian | 237,022,239 | 236,136,732 | 99.63% | 35,281,847,687 | 12.7929 | Our focus |
| SRR934420, SRR934432 | PRJNA210519 | SAMN02225750 | Hanwoo | 805,201,356 | 802,132,464 | 99.62% | 77,164,719,393 | 27.9749X | East Asian taurine |
| ERR4350687 | PRJEB39352 | SAMEA7057640 | Sahiwal | 741,422,896 | 733,895,708 | 98.98% | 102,479,530,462 | 37.1714X | Indian indicine |
| ERR4336349 | PRJEB39352 | SAMEA7057641 | Sahiwal | 670,422,823 | 649,329,231 | 96.85% | 90,105,424,160 | 32.6823XX | Indian indicine |
| ERR4336437 | PRJEB39352 | SAMEA7057642 | Sahiwal | 775,763,909 | 770,120,393 | 99.27% | 107,631,065,140 | 39.0377XX | Indian indicine |
| SRR12632083 | PRJNA658727 | SAMN16123981 | Sahiwal | 184,516,020 | 183,554,384 | 99.48% | 26,576,679,116 | 9.6391X | Indian indicine |
| SRR12632085 | PRJNA658727 | SAMN16123979 | Sahiwal | 262,070,124 | 260,659,325 | 99.46% | 37,710,556,516 | 13.6776X | Indian indicine |
| SRR12632109 | PRJNA658727 | SAMN16123957 | Sahiwal | 187,293,736 | 186,272,170 | 99.45% | 27,168,789,982 | 9.8544X | Indian indicine |
| SRR12632110 | PRJNA658727 | SAMN16123956 | Sahiwal | 237,786,925 | 236,335,498 | 99.39% | 34,213,200,956 | 12.409X | Indian indicine |
| SRR12632111 | PRJNA658727 | SAMN16123955 | Sahiwal | 193,883,504 | 192,894,384 | 99.49% | 27,819,339,473 | 10.0902X | Indian indicine |
| SRR13114524 | PRJNA658727 | SAMN16085988 | Sahiwal | 291,217,816 | 290,358,264 | 99.7% | 28,559,849,715 | 10.3572X | Indian indicine |
| SRR13114535 | PRJNA658727 | SAMN16085987 | Sahiwal | 296,151,044 | 294,387,119 | 99.4% | 29,078,633,684 | 10.5454X | Indian indicine |
| SRR13114546 | PRJNA658727 | SAMN16085986 | Sahiwal | 291,759,595 | 271,115,232 | 92.92% | 26,762,718,886 | 9.7056X | Indian indicine |
| SRR13114547 | PRJNA658727 | SAMN16085985 | Sahiwal | 137,300,868 | 136,559,465 | 99.46% | 13,535,309,968 | 4.9085X | Indian indicine |
| SRR10809648 | PRJNA598339 | SAMN13703163 | MongoliaMongolian | 234,683,544 | 233,972,667 | 99.70% | 34,367,256,677 | 12.4614X | Another population of Mongolian cattle |
| SRR10809649 | PRJNA598339 | SAMN13703162 | MongoliaMongolian | 254,022,202 | 253,284,342 | 99.71% | 37,299,876,305 | 13.524X | Another population of Mongolian cattle |
| SRR10809650 | PRJNA598339 | SAMN13703161 | MongoliaMongolian | 175,219,222 | 174,397,289 | 99.53% | 25,533,106,789 | 9.2576X | Another population of Mongolian cattle |
| SRR10809651 | PRJNA598339 | SAMN13703160 | InnerMongoliaMongolian | 297,079,784 | 296,284,874 | 99.73% | 29,348,544,177 | 10.6403X | Another population of Mongolian cattle |
| SRR10809652 | PRJNA598339 | SAMN137031590 | InnerMongoliaMongolian | 295,268,847 | 294,457,016 | 99.73% | 29,183,479,746 | 10.5803X | Another population of Mongolian cattle |
| SRR10809653 | PRJNA598339 | SAMN13703157 | InnerMongoliaMongolian | 267,301,178 | 266,378,224 | 99.65% | 26,361,186,898 | 9.5572X | Another population of Mongolian cattle |
| SRR10809654 | PRJNA598339 | SAMN13703164 | MongoliaMongolian | 209,171,064 | 208,300,822 | 99.58% | 30,473,440,357 | 11.0491X | Another population of Mongolian cattle |
| SRR10809655 | PRJNA598339 | SAMN13703158 | InnerMongoliaMongolian | 264,378,752 | 263,843,247 | 99.80% | 26,201,878,999 | 9.4993X | Another population of Mongolian cattle |
| SRR10809656 | PRJNA598339 | SAMN13703170 | MongoliaMongolian | 245,736,647 | 245,114,248 | 99.75% | 36,004,606,359 | 13.0547X | Another population of Mongolian cattle |
| SRR10809657 | PRJNA598339 | SAMN13703169 | MongoliaMongolian | 219,698,729 | 219,220,346 | 99.78% | 32,351,265,982 | 11.7296X | Another population of Mongolian cattle |
| SRR10809658 | PRJNA598339 | SAMN13703168 | MongoliaMongolian | 214,062,513 | 213,360,147 | 99.67% | 31,329,420,821 | 11.36X | Another population of Mongolian cattle |
| SRR10809659 | PRJNA598339 | SAMN13703167 | MongoliaMongolian | 207,722,551 | 207,338,576 | 99.82% | 30,524,536,457 | 11.0674X | Another population of Mongolian cattle |
| SRR10809660 | PRJNA598339 | SAMN13703166 | MongoliaMongolian | 183,897,762 | 183,318,476 | 99.68% | 26,957,019,379 | 9.7743X | Another population of Mongolian cattle |
| SRR10809661 | PRJNA598339 | SAMN13703165 | MongoliaMongolian | 180,390,958 | 179,856,802 | 99.70% | 26,429,830,995 | 9.5829X | Another population of Mongolian cattle |
| SRR10809662 | PRJNA598339 | SAMN13703156 | InnerMongoliaMongolian | 297,497,535 | 296,426,888 | 99.64% | 29,319,404,294 | 10.6296X | Another population of Mongolian cattle |
| SRR10809663 | PRJNA598339 | SAMN13703155 | InnerMongoliaMongolian | 299,620,718 | 298,511,424 | 99.63% | 29,498,948,855 | 10.6947X | Another population of Mongolian cattle |
| SRR10992774 | PRJNA604048 | SAMN13949656 | Muturu | 168,058,917 | 167,580,150 | 99.72% | 16,562,532,470 | 6.0048X | African taurine |
| SRR10992775 | PRJNA604048 | SAMN13949655 | Muturu | 183,647,726 | 183,297,923 | 99.81% | 18,293,969,849 | 6.6325X | African taurine |
| SRR10992776 | PRJNA604048 | SAMN13949654 | Muturu | 144,101,165 | 143,689,437 | 99.71% | 13,904,041,594 | 5.0411X | African taurine |
| SRR10992777 | PRJNA604048 | SAMN13949653 | Muturu | 147,747,888 | 147,349,159 | 99.73% | 14,705,144,961 | 5.3314X | African taurine |
| SRR14879644 | PRJNA737584 | SAMN19802773 | InnerMongoliaMongolian | 467,724,589 | 466,329,215 | 99.70% | 66,740,644,344 | 24.1982X | Another population of Mongolian cattle |
| SRR14879645 | PRJNA737584 | SAMN19802772 | InnerMongoliaMongolian | 487,920,626 | 486,409,036 | 99.69% | 69,687,097,902 | 25.2664X | Another population of Mongolian cattle |
| SRR14879646 | PRJNA737584 | SAMN19802771 | InnerMongoliaMongolian | 646,090,904 | 643,538,367 | 99.60% | 94,275,114,803 | 34.183X | Another population of Mongolian cattle |
| SRR14879647 | PRJNA737584 | SAMN19802770 | Leiqiong | 503,639,482 | 501,823,972 | 99.64% | 71,222,000,963 | 25.8352X | Chinese indicine |
| SRR14879648 | PRJNA737584 | SAMN19802769 | Leiqiong | 469,414,840 | 465,602,935 | 99.19% | 66,519,634,388 | 24.1296X | Chinese indicine |
| SRR14879650 | PRJNA737584 | SAMN19802767 | Leiqiong | 455,677,761 | 453,127,980 | 99.44% | 64,599,158,169 | 23.4332X | Chinese indicine |
| SRR14879659 | PRJNA737584 | SAMN19802766 | Leiqiong | 582,774,905 | 578,305,526 | 99.23% | 84,903,071,189 | 30.7986X | Chinese indicine |
| SRR14879667 | PRJNA737584 | SAMN19802776 | InnerMongoliaMongolian | 492,982,181 | 490,488,036 | 99.49% | 71,917,638,321 | 26.0762X | Another population of Mongolian cattle |
| SRR14879668 | PRJNA737584 | SAMN19802775 | InnerMongoliaMongolian | 476,133,499 | 474,709,985 | 99.70% | 68,036,876,173 | 24.6682X | Another population of Mongolian cattle |
| SRR14879669 | PRJNA737584 | SAMN19802774 | InnerMongoliaMongolian | 517,509,819 | 515,609,556 | 99.63% | 75,810,414,581 | 27.4869X | Another population of Mongolian cattle |
| SRR14879670 | PRJNA737584 | SAMN19802765 | Leiqiong | 456,435,807 | 453,214,250 | 99.29% | 66,538,048,790 | 24.1369X | Chinese indicine |
| SRR14879671 | PRJNA737584 | SAMN19802764 | Leiqiong | 506,848,123 | 499,023,026 | 98.46% | 73,386,367,620 | 26.6176X | Chinese indicine |
| SRR4280085 | PRJNA343262 | SAMN05788510 | Angus | 359,438,309 | 358,676,442 | 99.79% | 34,629,903,064 | 12.5546X | European taurine |
| SRR4280169 | PRJNA343262 | SAMN05788530 | Angus | 330,409,591 | 328,480,909 | 99.42% | 30,723,827,083 | 11.1384X | European taurine |
| SRR5507188 | PRJNA379859 | SAMN06699070 | Leiqiong | 209,943,696 | 208,287,447 | 99.21% | 29,523,132,335 | 10.7097X | Chinese indicine |
| SRR5507189 | PRJNA379859 | SAMN06699069 | Leiqiong | 222,010,454 | 219,544,200 | 98.89% | 30,952,972,161 | 11.2283X | Chinese indicine |
| SRR5507190 | PRJNA379859 | SAMN06699068 | Leiqiong | 229,795,413 | 227,262,967 | 98.90% | 32,013,450,926 | 11.6128X | Chinese indicine |
| SRR5507252 | PRJNA379859 | SAMN06698994 | Kazakh | 187,647,620 | 186,796,833 | 99.55% | 26,574,586,676 | 9.6349X | Another cattle breed of Xinjiang |
| SRR5507253 | PRJNA379859 | SAMN06698993 | Kazakh | 218,750,476 | 217,951,484 | 99.63% | 30,983,527,038 | 11.2335X | Another cattle breed of Xinjiang |
| SRR5507254 | PRJNA379859 | SAMN06698992 | Kazakh | 192,475,877 | 191,927,493 | 99.72% | 27,290,829,134 | 9.8946X | Another cattle breed of Xinjiang |
| SRR5507255 | PRJNA379859 | SAMN06698991 | Kazakh | 198,400,177 | 197,446,274 | 99.52% | 28,125,579,017 | 10.1973X | Another cattle breed of Xinjiang |
| SRR5507256 | PRJNA379859 | SAMN06698990 | Kazakh | 221,110,398 | 220,419,558 | 99.69% | 31,512,282,868 | 11.4252X | Another cattle breed of Xinjiang |
| SRR5507257 | PRJNA379859 | SAMN06698989 | Kazakh | 215,437,244 | 214,728,027 | 99.67% | 30,666,947,679 | 11.1188X | Another cattle breed of Xinjiang |
| SRR5507258 | PRJNA379859 | SAMN06698988 | Kazakh | 236,929,692 | 236,373,989 | 99.77% | 34,461,156,126 | 12.4947X | Another cattle breed of Xinjiang |
| SRR5507259 | PRJNA379859 | SAMN06698987 | Kazakh | 223,423,300 | 222,839,631 | 99.74% | 32,570,397,007 | 11.8088X | Another cattle breed of Xinjiang |
| SRR5507260 | PRJNA379859 | SAMN06698986 | Kazakh | 240,483,255 | 239,876,683 | 99.75% | 35,082,337,292 | 12.7195X | Another cattle breed of Xinjiang |
| SRR5507261 | PRJNA379859 | SAMN06698985 | XinjiangMongolian | 201,829,529 | 201,098,219 | 99.64% | 28,827,853,446 | 10.4522X | Our focus |
| SRR5507262 | PRJNA379859 | SAMN06698984 | XinjiangMongolian | 227,991,252 | 227,177,282 | 99.64% | 32,561,949,405 | 11.8058X | Our focus |
| SRR5507263 | PRJNA379859 | SAMN06698983 | XinjiangMongolian | 226,850,600 | 226,089,203 | 99.66% | 32,408,708,395 | 11.7509X | Our focus |
| SRR5507264 | PRJNA379859 | SAMN06698982 | XinjiangMongolian | 225,962,002 | 225,179,213 | 99.65% | 32,226,818,419 | 11.6847X | Our focus |
| SRR5507265 | PRJNA379859 | SAMN06698981 | XinjiangMongolian | 238,050,819 | 237,156,501 | 99.62% | 34,018,082,424 | 12.3339X | Our focus |
| SRR5507266 | PRJNA379859 | SAMN06698980 | XinjiangMongolian | 218,863,370 | 218,107,499 | 99.65% | 31,775,287,950 | 11.5213X | Our focus |
| SRR5507267 | PRJNA379859 | SAMN06698979 | XinjiangMongolian | 215,024,886 | 214,507,515 | 99.76% | 31,344,590,122 | 11.365X | Our focus |
| SRR5630644 | PRJNA386202 | SAMN07135498 | Muturu | 126,482,436 | 124,694,360 | 98.59% | 17,996,180,328 | 6.5248X | African taurine |
| SRR5630646 | PRJNA386202 | SAMN07135496 | Muturu | 124,141,958 | 121,132,633 | 97.58% | 17,528,300,662 | 6.3552X | African taurine |
| SRR5630647 | PRJNA386202 | SAMN07135495 | Muturu | 158,308,978 | 156,325,281 | 98.75% | 22,665,221,460 | 8.2176X | African taurine |
| SRR5630648 | PRJNA386202 | SAMN07135494 | Muturu | 180,764,075 | 176,874,274 | 97.85% | 25,484,957,591 | 9.2399X | African taurine |
| SRR5630649 | PRJNA386202 | SAMN07135493 | Muturu | 190,483,563 | 187,345,148 | 98.35% | 27,030,373,220 | 9.8001X | African taurine |
| SRR5630650 | PRJNA386202 | SAMN07135492 | Muturu | 180,384,389 | 178,843,129 | 99.15% | 25,433,502,555 | 9.2216X | African taurine |
| SRR5630651 | PRJNA386202 | SAMN07135491 | Muturu | 158,399,568 | 157,138,550 | 99.20% | 22,284,950,861 | 8.08X | African taurine |
| SRR5630652 | PRJNA386202 | SAMN07135500 | Muturu | 121,478,209 | 119,036,309 | 97.99% | 17,256,114,236 | 6.2565X | African taurine |
| SRR5630653 | PRJNA386202 | SAMN07135499 | Muturu | 161,256,233 | 157,826,920 | 97.87% | 22,793,631,525 | 8.2642X | African taurine |
| SRR6234776 | PRJNA396672 | SAMN07430999 | InnerMongoliaMongolian | 351,042,196 | 350,209,620 | 99.76% | 33,628,315,441 | 12.1913X | Another population of Mongolian cattle |
| SRR6234777 | PRJNA396672 | SAMN07430998 | InnerMongoliaMongolian | 349,403,074 | 348,278,977 | 99.68% | 33,359,811,364 | 12.0939X | Another population of Mongolian cattle |
| SRR8587810 | PRJNA343262 | SAMN10940449 | Angus | 343,862,647 | 342,719,033 | 99.67% | 32,388,105,453 | 11.7417X | European taurine |
| SRR8587861 | PRJNA343262 | SAMN10940504 | Angus | 330,937,364 | 329,304,765 | 99.51% | 30,749,630,265 | 11.1477X | European taurine |
| SRR8587866 | PRJNA343262 | SAMN10940491 | Angus | 345,612,002 | 343,532,179 | 99.40% | 32,822,042,841 | 11.8989X | European taurine |
| SRR8587867 | PRJNA343262 | SAMN10940488 | Angus | 319,348,401 | 318,238,714 | 99.65% | 29,645,899,889 | 10.7473X | European taurine |
| SRR8587873 | PRJNA343262 | SAMN10940490 | Angus | 341,166,786 | 340,154,644 | 99.70% | 32,257,777,706 | 11.6942X | European taurine |
| SRR8587876 | PRJNA343262 | SAMN10940468 | Angus | 337,116,781 | 336,028,494 | 99.68% | 31,434,193,416 | 11.3956X | European taurine |
| SRR8587987 | PRJNA343262 | SAMN10940502 | Angus | 273,635,849 | 266,375,177 | 97.35% | 24,580,732,408 | 8.9112X | European taurine |
| SRR8588000 | PRJNA343262 | SAMN10940471 | Angus | 334,363,035 | 333,265,870 | 99.67% | 31,465,705,946 | 11.4072X | European taurine |
| SRR8588012 | PRJNA343262 | SAMN10940477 | Angus | 342,559,695 | 341,738,286 | 99.76% | 32,657,139,985 | 11.839X | European taurine |
| SRR8588027 | PRJNA343262 | SAMN10940484 | Angus | 346,116,237 | 344,729,247 | 99.60% | 32,693,603,157 | 11.8524X | European taurine |
| SRR8588044 | PRJNA343262 | SAMN10940455 | Angus | 332,677,297 | 331,553,348 | 99.66% | 31,099,359,103 | 11.2745X | European taurine |
| SRR8588089 | PRJNA343262 | SAMN10940516 | Angus | 344,388,491 | 342,837,306 | 99.55% | 32,366,795,081 | 11.7338X | European taurine |
| SRR8588091 | PRJNA343262 | SAMN10940515 | Angus | 337,047,178 | 332,907,456 | 98.77% | 31,830,887,392 | 11.5396X | European taurine |
| SRR8588092 | PRJNA343262 | SAMN10940515 | Angus | 323,520,488 | 319,395,516 | 98.72% | 30,154,739,137 | 10.932X | European taurine |
| SRR8588093 | PRJNA343262 | SAMN10940514 | Angus | 341,130,452 | 246,232,239 | 72.18% | 23,526,465,777 | 8.529X | European taurine |
| SRR8588099 | PRJNA343262 | SAMN10940475 | Angus | 340,680,941 | 339,897,630 | 99.77% | 32,301,691,935 | 11.7102X | European taurine |
| SRR8588117 | PRJNA343262 | SAMN10940446 | Angus | 333,353,691 | 332,504,980 | 99.75% | 31,776,537,795 | 11.5198X | European taurine |
| SRR8588118 | PRJNA343262 | SAMN10940446 | Angus | 333,089,468 | 332,128,429 | 99.71% | 31,501,531,223 | 11.4201X | European taurine |
| SRR8588157 | PRJNA343262 | SAMN10940492 | Angus | 338,166,981 | 337,228,252 | 99.72% | 31,808,071,484 | 11.5311X | European taurine |
| SRR8588164 | PRJNA343262 | SAMN10940464 | Angus | 330,387,885 | 329,441,790 | 99.71% | 30,965,870,922 | 11.226X | European taurine |
| SRR8588172 | PRJNA343262 | SAMN10940462 | Angus | 336,258,610 | 335,167,672 | 99.68% | 31,598,976,206 | 11.4553X | European taurine |
| SRR8588261 | PRJNA343262 | SAMN10940524 | Angus | 339,134,787 | 334,389,538 | 98.60% | 31,731,074,848 | 11.5033X | European taurine |
| SRR926762 | PRJNA210523 | SAMN02225723 | Hanwoo | 310,375,295 | 308,834,682 | 99.50% | 29,948,473,013 | 10.8576X | East Asian taurine |
| SRR934395 | PRJNA210523 | SAMN02225724 | Hanwoo | 277,798,309 | 276,662,308 | 99.59% | 26,933,649,288 | 9.7645X | East Asian taurine |
| SRR934396 | PRJNA210523 | SAMN02225725 | Hanwoo | 335,101,487 | 333,831,553 | 99.62% | 31,591,757,184 | 11.4532X | East Asian taurine |
| SRR934397 | PRJNA210523 | SAMN02225726 | Hanwoo | 240,809,703 | 239,967,921 | 99.65% | 23,339,239,368 | 8.4613X | East Asian taurine |
| SRR934399 | PRJNA210523 | SAMN02225728 | Hanwoo | 313,440,378 | 312,218,980 | 99.61% | 29,723,281,663 | 10.7758X | East Asian taurine |
| SRR934400 | PRJNA210523 | SAMN02225729 | Hanwoo | 364,049,980 | 362,789,546 | 99.65% | 34,322,923,570 | 12.4434X | East Asian taurine |
| SRR934401 | PRJNA210523 | SAMN02225732 | Hanwoo | 395,262,943 | 393,941,966 | 99.67% | 37,989,397,967 | 13.7727X | East Asian taurine |
| SRR934402 | PRJNA210523 | SAMN02225733 | Hanwoo | 326,286,694 | 322,806,485 | 98.93% | 30,996,415,103 | 11.2379X | East Asian taurine |
| SRR934403 | PRJNA210523 | SAMN02225730 | Hanwoo | 295,727,417 | 294,432,480 | 99.56% | 28,726,686,293 | 10.4146X | East Asian taurine |
| SRR934404 | PRJNA210523 | SAMN02225731 | Hanwoo | 345,872,526 | 344,651,464 | 99.65% | 32,549,014,075 | 11.8003X | East Asian taurine |
| SRR934415 | PRJNA210519 | SAMN02225744 | Hanwoo | 387,228,311 | 385,712,097 | 99.61% | 37,174,404,864 | 13.4773X | East Asian taurine |
| SRR934416 | PRJNA210519 | SAMN02225745 | Hanwoo | 410,918,985 | 409,307,905 | 99.61% | 39,185,801,293 | 14.2062X | East Asian taurine |
| SRR934417 | PRJNA210519 | SAMN02225746 | Hanwoo | 382,854,001 | 381,084,889 | 99.54% | 36,608,963,065 | 13.2723X | East Asian taurine |
| SRR934418 | PRJNA210519 | SAMN02225747 | Hanwoo | 402,614,425 | 400,640,831 | 99.51% | 38,299,965,833 | 13.8851X | East Asian taurine |
| SRR934419 | PRJNA210519 | SAMN02225748 | Hanwoo | 373,967,299 | 372,508,054 | 99.61% | 35,921,336,530 | 13.0228X | East Asian taurine |
| SRR934433 | PRJNA210519 | SAMN02225751 | Hanwoo | 365,564,281 | 361,570,418 | 98.91% | 34,919,418,631 | 12.6596X | East Asian taurine |
| SRR934434 | PRJNA210519 | SAMN02225752 | Hanwoo | 371,532,631 | 370,155,537 | 99.63% | 35,722,600,292 | 12.9509X | East Asian taurine |
| SRR934435 | PRJNA210519 | SAMN02225753 | Hanwoo | 337,067,603 | 335,793,255 | 99.62% | 32,591,957,500 | 11.816X | East Asian taurine |
| SRR934436 | PRJNA210519 | SAMN02225754 | Hanwoo | 334,402,634 | 333,218,133 | 99.65% | 32,292,563,302 | 11.7079X | East Asian taurine |
| SRR934437 | PRJNA210519 | SAMN02225755 | Hanwoo | 348,140,316 | 346,931,780 | 99.65% | 33,603,647,765 | 12.1826X | East Asian taurine |

**Supplementary Table 2** Functional classification of the detected SNPs.

| Functional classification | Number of SNPs |
| --- | --- |
| UTR3 | 444921 |
| UTR5 | 157873 |
| UTR5;UTR3 | 389 |
| downstream | 391386 |
| exonic | 700853 |
| exonic;splicing | 156 |
| intergenic | 36527635 |
| intronic | 22902643 |
| ncRNA_exonic | 7158 |
| ncRNA_intronic | 13808 |
| ncRNA_splicing | 12 |
| splicing | 1954 |
| upstream | 365395 |
| upstream;downstream | 13950 |

**Supplementary Table 3** Functional classification of the exonic SNPs.

| Functional classification | Number of SNPs |
| --- | --- |
| nonsynonymous | 255920 |
| stopgain | 3065 |
| stoploss | 327 |
| synonymous | 425549 |
| unknown | 16148 |

**Supplementary Table 4** list of selected regions in Xinjiang Mongolian cattle.

| Chr | Start | End | π ratio | *F_ST_* | XP-EHH | Genes |
| --- | --- | --- | --- | --- | --- | --- |
| 1 | 4200001 | 4270000 | 1.845685074 | NS | 2.19 | TIAM1 |
| 1 | 27180001 | 27270000 | 1.67530782 | 0.191473 | NS | ROBO1 |
| 1 | 65560001 | 65730000 | 1.745028498 | 0.171733 | NS | STXBP5L |
| 2 | 16180001 | 16430000 | 1.942156487 | 0.119901 | NS |  |
| 2 | 17120001 | 17250000 | 1.792558049 | NS | 2.77 | ZNF385B |
| 2 | 18980001 | 19110000 | 1.624566309 | NS | 2.71 | PDE11A |
| 2 | 80120001 | 80230000 | 2.017664455 | NS | 2.22 |  |
| 2 | 81420001 | 81690000 | 1.948478437 | 0.171868 | 3.73 |  |
| 2 | 83020001 | 83190000 | 1.826892228 | 0.186457 | 3.97 |  |
| 2 | 110220001 | 110330000 | 1.683091888 | NS | 2.96 |  |
| 2 | 119580001 | 119770000 | 1.770597222 | NS | 2.84 | NPPC\|DIS3L2 |
| 2 | 128160001 | 128290000 | 1.666772464 | NS | 2.71 | SRRM1\|CLIC4 |
| 3 | 9060001 | 9490000 | 1.873735885 | NS | 3.39 | LOC104971425\|NHLH1\|CASQ1\|CD84\|VANGL2\|NCSTN\|COPA\|PEX19\|PEA15\|ATP1A4\|SLAMF6\|DCAF8 |
| 3 | 23520001 | 23610000 | 1.736341124 | NS | 2.6 | HMGCS2\|PHGDH |
| 3 | 24420001 | 24510000 | 1.603574333 | 0.119726 | 3.12 |  |
| 3 | 53920001 | 54010000 | 1.768266499 | NS | 2.22 | LOC786372\|LOC107131333 |
| 3 | 54040001 | 54130000 | 1.821312001 | 0.117425 | NS | GBP6\|GBP5 |
| 3 | 72080001 | 72290000 | 2.169602441 | NS | 2.95 |  |
| 3 | 83180001 | 83290000 | 2.258827647 | NS | 2.27 | KANK4 |
| 3 | 85300001 | 85390000 | 1.811561517 | NS | 3.13 |  |
| 3 | 85820001 | 86030000 | 1.809239809 | NS | 3.68 | LOC521656\|LOC107132327\|LOC530929\|LOC511936 |
| 4 | 7820001 | 7930000 | 1.636504566 | NS | 3.03 |  |
| 4 | 14200001 | 14350000 | 1.616381738 | NS | 3.09 |  |
| 5 | 18620001 | 18730000 | 2.356019472 | NS | 3.14 |  |
| 5 | 18760001 | 18990000 | 1.75371732 | NS | 2.62 |  |
| 5 | 51660001 | 51730000 | 1.780897071 | NS | 2.49 | FAM19A2 |
| 5 | 68660001 | 68830000 | 2.209948334 | NS | 2.24 | APPL2\|C5H12orf75\|WASHC4 |
| 6 | 9540001 | 10010000 | 1.911940685 | NS | 3.95 |  |
| 6 | 14660001 | 14770000 | 2.268042481 | NS | 2.58 |  |
| 6 | 38880001 | 39130000 | 2.485168488 | NS | 3.4 |  |
| 6 | 39460001 | 39970000 | 2.247693291 | NS | 3.1 | SLIT2 |
| 6 | 39980001 | 40330000 | 2.911576859 | NS | 2.42 | PACRGL\|SLIT2\|KCNIP4 |
| 6 | 43600001 | 43870000 | 2.660633802 | NS | 2.92 | PPARGC1A |
| 6 | 51300001 | 51370000 | 1.756143759 | NS | 2.23 |  |
| 6 | 54980001 | 55090000 | 2.17821879 | NS | 2.31 |  |
| 6 | 60240001 | 60330000 | 2.217051296 | NS | 2.75 | LIMCH1 |
| 6 | 66180001 | 66290000 | 1.851974765 | NS | 2.28 | ATP10D\|CORIN |
| 7 | 4680001 | 4770000 | 1.7122715 | NS | 2.23 | ISYNA1\|SSBP4\|ELL |
| 7 | 36180001 | 36290000 | 1.71015244 | NS | 3.59 |  |
| 7 | 80720001 | 81010000 | 3.349094683 | NS | 2.82 | MSH3\|RASGRF2\|DHFR |
| 7 | 87120001 | 87390000 | 2.570151571 | NS | 2.44 |  |
| 8 | 38400001 | 38630000 | 3.602338118 | 0.115282 | 2.74 | TPD52L3\|IL33 |
| 8 | 43380001 | 43570000 | 3.670723422 | NS | 2.48 | DMRT2 |
| 8 | 72560001 | 72810000 | 2.275430275 | NS | 2.96 | GNRH1\|DOCK5\|KCTD9 |
| 8 | 106100001 | 106370000 | 1.890776471 | 0.171548 | 4.08 | ASTN2 |
| 8 | 107320001 | 107430000 | 1.668053539 | NS | 2.56 |  |
| 8 | 108700001 | 108910000 | 1.91300167 | NS | 2.74 | BRINP1 |
| 9 | 30920001 | 30990000 | 2.232916366 | NS | 2.29 |  |
| 9 | 31040001 | 31150000 | 1.875759354 | NS | 2.22 |  |
| 9 | 31160001 | 31250000 | 1.703359505 | NS | 2.32 |  |
| 9 | 78140001 | 78310000 | 2.356947704 | NS | 2.15 |  |
| 10 | 14000001 | 14170000 | 1.790475619 | NS | 3.02 | SMAD3\|AAGAB\|IQCH |
| 10 | 19180001 | 19330000 | NS | 0.116616 | 2.41 | PARP6\|CELF6\|TMEM202\|HEXA |
| 10 | 26560001 | 26770000 | 1.904098456 | NS | 4.33 | RNASE10\|PNP\|LOC790312\|PIP4P1\|OSGEP\|KLHL33\|APEX1\|PARP2\|TEP1 |
| 10 | 70920001 | 71030000 | 1.639206024 | NS | 2.37 |  |
| 11 | 26640001 | 26690000 | NS | 0.120886 | 2.57 | PPM1B |
| 11 | 92720001 | 92810000 | NS | 0.114213 | 2.15 | TTLL11 |
| 11 | 102920001 | 102990000 | NS | 0.132038 | 2.8 | SPACA9\|TSC1 |
| 12 | 40860001 | 40970000 | 1.747350866 | 0.119936 | NS |  |
| 12 | 85460001 | 85510000 | 1.706336281 | 0.122454 | NS | ARHGEF7 |
| 13 | 29320001 | 29430000 | 1.984856846 | 0.118646 | 2.53 |  |
| 14 | 6400001 | 6510000 | 1.62890753 | NS | 2.7 | KHDRBS3 |
| 14 | 33920001 | 34150000 | 3.891166631 | NS | 2.17 | NCOA2 |
| 14 | 46980001 | 47230000 | 1.773571715 | NS | 2.75 | SLC30A8 |
| 15 | 51260001 | 51430000 | NS | 0.121058 | 3.25 | LOC407145\|LOC100336836\|NUP98\|CHRNA10\|LOC112441549\|PGAP2\|ART1\|LOC112441636 |
| 16 | 120001 | 170000 | 3.516315831 | 0.278748 | NS |  |
| 16 | 26020001 | 26090000 | 2.01576396 | 0.173605 | NS | HHIPL2\|TAF1A |
| 16 | 33280001 | 33370000 | 1.796248139 | NS | 2.6 | AKT3 |
| 16 | 61020001 | 61290000 | 1.85397061 | 0.198873 | 3.41 | TOR1AIP1\|FAM163A\|TOR1AIP2\|CEP350 |
| 18 | 31420001 | 31830000 | 1.795862087 | NS | 3.06 |  |
| 18 | 61100001 | 61230000 | 1.907296999 | NS | 2.57 | LOC112442379\|LOC513941\|LOC112442378\|LOC527385\|LOC528332\|LOC617141 |
| 20 | 3660001 | 3890000 | 2.988211563 | NS | 2.88 | FBXW11\|STK10 |
| 20 | 20260001 | 20570000 | 1.881904751 | 0.122755 | 4.2 | RAB3C\|PDE4D |
| 20 | 30580001 | 30670000 | 1.686917532 | NS | 2.76 | FGF10 |
| 21 | 6100001 | 6310000 | 2.000722155 | NS | 2.87 | CERS3 |
| 21 | 10320001 | 10510000 | 1.682079467 | 0.117193 | 3.1 |  |
| 22 | 46460001 | 46530000 | NS | 0.132101 | 2.35 | CACNA2D3 |
| 23 | 11520001 | 11630000 | 1.743348771 | 0.181836 | 3.2 | MDGA1 |
| 23 | 24060001 | 24150000 | 1.720271711 | NS | 2.29 | PKHD1 |
| 24 | 8540001 | 8690000 | 2.333436827 | NS | 2.3 | LOC100337355 |
| 24 | 13180001 | 13230000 | 1.665143404 | NS | 2.26 |  |
| 24 | 15220001 | 15290000 | 2.280077718 | NS | 2.22 |  |
| 24 | 19840001 | 20350000 | 1.944657475 | 0.207493 | 3.2 | TPGS2\|FHOD3\|KIAA1328 |
| 24 | 56780001 | 56950000 | 1.635294066 | NS | 2.95 | FECH\|NARS\|ATP8B1 |
| 26 | 920001 | 1190000 | 1.856502677 | NS | 2.76 |  |
| 26 | 38260001 | 38410000 | 2.258307169 | 0.126653 | 3.15 | RAB11FIP2 |
| 26 | 45240001 | 45310000 | 1.687474278 | NS | 2.59 | EDRF1\|UROS |
| 26 | 45820001 | 45890000 | 1.998255156 | NS | 2.76 | ADAM12 |
| 26 | 50260001 | 50370000 | 1.863920066 | 0.184072 | NS | TCERG1L |
| 27 | 9500001 | 9610000 | 3.873109247 | NS | 2.32 |  |
| 27 | 14140001 | 14290000 | 1.771254261 | NS | 2.16 | LOC112444630\|CDKN2AIP\|LOC536739\|WWC2 |
| 27 | 21300001 | 21690000 | 2.109171288 | 0.137045 | 3.29 | TUSC3 |
| 29 | 35440001 | 35530000 | 1.653337206 | NS | 2.58 | NTM |
| 29 | 48960001 | 49050000 | 1.705365888 | NS | 2.35 | KCNQ1 |

**Supplementary Table 5** The top ten significant GO terms from the enrichment analysis of selected candidate genes

| Category | Term | Count | PValue | Genes | FDR |
| --- | --- | --- | --- | --- | --- |
| MF | GO:0016712~oxidoreductase activity, acting on paired donors, with incorporation or reduction of molecular oxygen, reduced flavin or flavoprotein as one donor, and incorporation of one atom of oxygen | 4 | 3.73E-04 | LOC107132327, LOC521656, LOC530929, LOC511936 | 0.031713 |
| MF | GO:0051117~ATPase binding | 5 | 4.10E-04 | NCSTN, PEX19, PDE4D, TOR1AIP2, TOR1AIP1 | 0.031713 |
| MF | GO:0003950~NAD+ ADP-ribosyltransferase activity | 4 | 5.06E-04 | ART1, PARP6, PARP2, LOC407145 | 0.031713 |
| MF | GO:0008395~steroid hydroxylase activity | 4 | 7.89E-04 | LOC107132327, LOC521656, LOC530929, LOC511936 | 0.037097 |
| BP | GO:0048754~branching morphogenesis of an epithelial tube | 4 | 1.40E-04 | PKHD1, CLIC4, SLIT2, FGF10 | 0.065456 |
| BP | GO:0006471~protein ADP-ribosylation | 4 | 1.97E-04 | ART1, PARP6, LOC100336836, PARP2 | 0.065456 |
| MF | GO:0015189~L-lysine transmembrane transporter activity | 3 | 0.004519 | LOC617141, LOC112442378, LOC527385 | 0.133715 |
| MF | GO:0015181~arginine transmembrane transporter activity | 3 | 0.004519 | LOC617141, LOC112442378, LOC527385 | 0.133715 |
| MF | GO:0000064~L-ornithine transmembrane transporter activity | 3 | 0.004979 | LOC617141, LOC112442378, LOC527385 | 0.133715 |
| MF | GO:0005506~iron ion binding | 5 | 0.00642 | FECH, LOC107132327, LOC521656, LOC530929, LOC511936 | 0.150868 |

**Supplementary Table 6** The top ten significant KEGG pathways from the enrichment analysis of selected candidate genes

| Term | Count | PValue | Genes | FDR |
| --- | --- | --- | --- | --- |
| bta00591:Linoleic acid metabolism | 4 | 0.001945 | LOC107132327, LOC521656, LOC530929, LOC511936 | 0.365692 |
| bta04913:Ovarian steroidogenesis | 4 | 0.005516 | LOC107132327, LOC521656, LOC530929, LOC511936 | 0.518472 |
| bta00590:Arachidonic acid metabolism | 4 | 0.012706 | LOC107132327, LOC521656, LOC530929, LOC511936 | 0.796245 |
| bta04750:Inflammatory mediator regulation of TRP channels | 4 | 0.0253 | LOC107132327, LOC521656, LOC530929, LOC511936 | 1 |
| bta04726:Serotonergic synapse | 4 | 0.032377 | LOC107132327, LOC521656, LOC530929, LOC511936 | 1 |
| bta01100:Metabolic pathways | 16 | 0.034657 | CERS3, ISYNA1, FECH, LOC790312, PDE4D, UROS, HEXA, LOC530929, LOC511936, DHFR, PDE11A, PNP, LOC107132327, LOC521656, PHGDH, HMGCS2 | 1 |
| bta00230:Purine metabolism | 4 | 0.041158 | PDE11A, PNP, LOC790312, PDE4D | 1 |
| bta05417:Lipid and atherosclerosis | 5 | 0.047825 | LOC107132327, LOC521656, AKT3, LOC530929, LOC511936 | 1 |
| bta04261:Adrenergic signaling in cardiomyocytes | 4 | 0.060044 | KCNQ1, AKT3, ATP1A4, CACNA2D3 | 1 |
| bta04218:Cellular senescence | 4 | 0.065794 | SMAD3, FBXW11, AKT3, TSC1 | 1 |
